# Parent–child inpatient treatment for children with behavioural and emotional disorders: a multilevel analysis of within-subjects effects

**DOI:** 10.1186/s12888-015-0675-7

**Published:** 2015-11-16

**Authors:** Elena Ise, Sabine Schröder, Dieter Breuer, Manfred Döpfner

**Affiliations:** Department of Child and Adolescent Psychiatry and Psychotherapy, Medical Faculty, University of Cologne, Robert-Koch-Str. 10, Cologne, 50931 Germany; School for Child and Adolescent Psychotherapy at the University Hospital Cologne, Robert-Koch-Str. 10, Cologne, 50931 Germany

**Keywords:** Inpatient treatment, Parent–child interaction, Family treatment, Child and adolescent psychiatry, Parent training

## Abstract

**Background:**

The importance of parental involvement in child treatment is well-established. Several child psychiatric clinics have, therefore, set up inpatient family units where children and parents are both actively involved in the treatment. Unfortunately, evidence supporting the benefits of these units is sparse.

**Methods:**

We evaluated the effectiveness of inpatient treatment for families with severe parent–child interaction problems in a child psychiatric setting. Consecutive admissions to the parent–child ward (*N* = 66) were studied. A within-subjects design was used with four assessment points (baseline, admission, discharge, four-week follow-up). Outcome measures were 1) parent and teacher ratings of child behaviour, and 2) parent self-ratings of parenting practices, parental strains and parental mental health. Data were analyzed using multilevel modelling for longitudinal data (piecewise growth curve models).

**Results:**

All parent-rated measures improved significantly during the four-week treatment period (*d* = 0.4 – 1.3). These improvements were significantly greater than those observed during the four-week pre-admission period. In addition, benefits were maintained during the four-week follow-up period. Only parents’ self-efficacy in managing their child’s behaviour showed continued improvement during follow-up. Teacher ratings of children’s disruptive behaviour at school were stable during the pre-admission period and showed significant improvements at follow-up (*d* = 0.3 – 0.4).

**Conclusions:**

We conclude that parent–child inpatient treatment has positive effects on child and parent behaviour and mental health, and can therefore be recommended for children with behavioural and emotional disorders and severe parent–child interaction problems.

## Background

Inpatient treatment is an important component of child and adolescent mental health services. It is considered the most restrictive type of care [1] and admission should be reserved for children with severe psychiatric symptomatology. Until recently, the evidence base for child psychiatric inpatient treatment was weak due to a lack of methodologically sound studies [1, 2]. This situation has changed gradually over the past 15 years. A few multicenter follow-up studies [3, 4], randomized controlled trials [5] and several single-group studies [6, 7] have been published that demonstrate beneficial effects of child psychiatric inpatient treatment. In these studies, children were admitted to inpatient treatment without their parents, which is the predominant approach in child psychiatric inpatient treatment and clinicians focused on working directly with the admitted child, rather than on educating and training the parents. Inpatient treatment often includes parental guidance or family therapy, but in practice most parents attend relatively few sessions [6, 7].

The vast majority of cognitive-behavioural interventions in routine outpatient care, in contrast, employ family-based interventions as a key component [8, 9]. The effectiveness of parental involvement in child psychiatric outpatient treatment has been demonstrated in several clinical trials with different patient populations, including children with Attention Deficit-/Hyperactivity Disorder (ADHD; [10]), Oppositional Defiant and Conduct Disorders (ODD/CD; [11, 12]), Autism Spectrum Disorders (ASD; [13]) and emotional disorders like anxiety and depression [14]. For young children with disruptive behaviour disorders (ODD/CD, ADHD) parent training programmes that work directly with parents to modify their parenting behaviours are considered as a first line approach [12, 15] .

Effective components of parent training programmes include enhancing the parent–child relationship, teaching the use of time out, decreasing negative, harsh or inconsistent parenting practices and requiring parents to practice new parenting skills with their own child during training sessions [16, 17]. Most parent training programmes contain several effective elements. Parent–child Interaction Therapy (PCIT; [18]), for example, helps parents to modify their parenting behaviour via direct, in vivo coaching strategies. Parents are taught to play with their children in a positive and non-directive way and to apply behaviour management techniques such as using clear instructions and appropriate consequences for noncompliance. The positive outcomes of PCIT are well documented [19].

The German treatment manual THOP (Treatment Program for Hyperkinetic and Oppositional Problem Behaviour; [20]) combines parent management training including direct coaching of parent–child dyads, school-based interventions and child-focused interventions. It has been shown to be effective in outpatient settings over both the short and the long term [21–23]. It was also used successfully in the day treatment of preschool children with developmental delays and behaviour disorders [24] and, with adaptations, as an indicated prevention programme [25–28].

Despite these promising findings, it is important to keep in mind that outpatient parent training is not equally effective for all families. There is, for example, evidence that children from economically disadvantaged or single-parent families benefit less from parent training programmes than their peers [29]. In addition, attendance rates are often low [30, 31] and many children continue to show clinically meaningful behaviour problems after parent training [32], suggesting that some families need more intensive treatment. Children with severe behavioural problems, whose parents have low parenting skills, might benefit more from inpatient treatment of parent–child interaction problems that includes both parent-focused and child-focused interventions.

Several child psychiatric clinics have, therefore, set up inpatient family units where children and their parents are admitted together and both are involved in the treatment of the child’s problem behaviour (e.g., [33, 34]). However, to date, only a few studies have evaluated the effectiveness of parent–child inpatient treatment. Their results provide initial evidence that inpatient family units positively influence family functioning. For example, a Swedish multicentre study showed that inpatient treatment of the whole family resulted in improved family climate [35]. Another study evaluated changes in parenting dimensions and parental distress in families treated at child psychiatric family inpatient units in Norway [36]. The non-standardized treatment targeted family communication and mutual understanding, but it did not focus explicitly on the reduction of dysfunctional parenting practices or parental distress. Yet, mothers reported a significant decrease of symptoms of anxiety and depression at the 3-month and 12-month follow-up. Parents of children with attention disorders showed a significant increase in the parenting dimension warmth, but parents of children with emotional problems did not report a change in their level of warmth. The dimensions protectiveness and authoritarianism did not change significantly in any group.

To our knowledge, only one study has so far reported the effects of parent–child inpatient treatment in a child psychiatric setting on child behaviour outcomes. The study evaluated the outcome of an intensive multimodal inpatient family treatment programme in Melbourne, Australia, using archival data of 29 families [37]. Up to two families were admitted at a time for a stay of two to six weeks. The referred children showed severe emotional and behavioural problems and many parents had psychiatric problems of their own. Consistent with the studies cited above [35, 36], parents reported significant improvements in parent and family functioning. In addition, mothers and clinicians both reported a significant improvement in child behaviour (assessed with the Child Behaviour Checklist, CBCL; [38]) between admission and discharge. Whether these effects remained stable over time could not be addressed due to high attrition from baseline to follow-up (>50 %).

The goals of the present article are: (1) to describe the concept of an inpatient setting that provides treatment to both children and parents (parent–child ward) using evidence-based treatment manuals (e.g., THOP), and (2) to analyze treatment-related changes in child behaviour problems, parenting behaviour, parental self-efficacy, parental strains and parental mental health. Sixty-six consecutively admitted children aged 3–10 years participated in the effectiveness study. Child and parent outcomes were assessed at baseline (four weeks before admission), at admission to inpatient treatment, at discharge (after four weeks of inpatient treatment) and after a four-week follow-up period. Multilevel analysis was employed to investigate changes in outcomes over time. We expected that child and parent variables would be stable during the pre-admission waiting period, would show significant improvements during the treatment period, which would be stronger than changes during the waiting period, and would stabilize during the follow-up period.

## Methods

### Study design and data collection

This observation study of routine care used a single-group, within-subjects design (repeated measures) with participants serving as their own controls. The course of outcome measures was investigated during a four-week pre-admission waiting period, a four-week inpatient treatment period and a subsequent four-week follow-up period. Data were collected at four assessment points. The baseline assessment (T1) took place four weeks before admission to inpatient treatment. The pre-treatment assessment (T2) was conducted at admission. The post-treatment assessment (T3) was done at discharge, immediately after the four-week inpatient treatment period. The follow-up assessment (T4) was carried out four weeks after discharge. Parents were asked to participate in all measurement occasions. Teachers were asked to participate in the T1, the T2 and the T4 assessment. They were not asked to participate in the post-treatment (T3) assessment because the participating children did not attend their regular school during the treatment period. The study was approved by ethical committee of the Medical Faculty of the University of Cologne.

### Participants

**Fig. 1:**
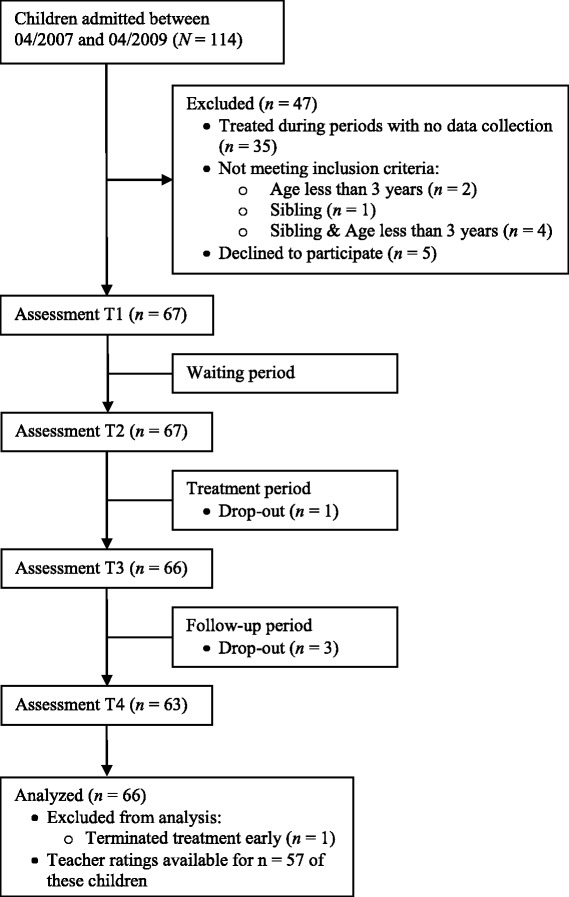
Flow of participants through the study

One hundred fourteen children were consecutively admitted to the parent–child ward of the Department of Child and Adolescent Psychiatry and Psychotherapy at the University Hospital of Cologne between 04/2007 and 04/2009. Due to practical reasons, data collection was not possible in children admitted from May to August 2007 (*N* = 18), in February and March 2008 (*N* = 9), in July 2008 (*N* = 4), or in November 2008 (*N* =4). Children treated during this time period (*N* = 35) could not be included in this study. Children aged less than 3 years (*N* = 6) and siblings of patients (i.e. not index patients, *N* = 1) were excluded. Seventy-two families were asked to participate in the study. Of these, sixty-seven (93 %) were willing to participate. Parental verbal informed consent was obtained before admission. One family terminated treatment early because of maternal psychiatric problems and was excluded from data analysis. Figure 1 depicts the flow of participants through the study. The final sample includes 66 children who were aged 3 to 10 years old (*M* = 6.9, *SD* = 1.7), had serious behavioural and/or emotional problems and showed severe parent–child interaction problems (clinical judgement of the treatment team). There were no further inclusion or exclusion criteria. Parent ratings were available for all 66 children. Teacher ratings were available for 57 of these children. Most children (*N* = 59, 89 %) were of normal intelligence (IQ ≥ 85), seven children (11 %) showed below-average cognitive abilities. The majority of children (60 %) had received outpatient psychotherapy of varying intensity prior to admission to the parent–child ward.

The sample comprises 17 girls (26 %) and 49 boys (74 %). Thirty-eight children (58 %) lived with both parents, 20 children (30 %) lived with their mother only, six children (9 %) lived with their mother and a stepfather and two children (3 %) lived with a foster family. Mothers were aged 22 to 50 years old (*N* = 66, *M* = 35.8, *SD* = 6.2). Fathers were slightly older (*N* = 60, 26 – 52 years old, *M* = 38.8, *SD* = 6.3). Fifty percent of mothers (*N* = 33) were working (part-time: *N* = 24, 36 %; full-time: *N* = 9, 14 %). Data on work status were available for 53 fathers. Most of them were working full-time (*N* = 45, 85 %), eight (15 %) were not in paid work.

All participants met criteria for one (*N* = 58) or two (*N* = 8) ICD-10 diagnoses. The most common diagnoses were hyperkinetic disorders with or without comorbid conduct disorder (F90, *N* = 36, 55 %), emotional disorders with onset specific to childhood (F93, *N* = 11, 17 %), other behavioural and emotional disorders with onset usually occurring in childhood and adolescence (F98, *N* = 8, 12 %), pervasive developmental disorders (F84, *N* = 5, 8 %), followed by conduct disorders (F91, *N* = 4, 6 %), mixed disorders of conduct and emotions (F92, *N* = 4, 6 %), reaction to severe stress and adjustment disorders (F43, *N* = 4, 6 %), disorders of social functioning with onset specific to childhood and adolescence (F94, *N* = 1, 2 %). Seventeen children (26 %) used stimulant medication for the treatment of ADHD symptoms at admission (T2). Four of these (6 % of the sample) stopped stimulant treatment during the treatment phase (between T2 and T3). Forty-nine children (74 %) did not use stimulant medication at admission. Twelve of these (18 % of sample) started stimulant treatment during the treatment phase. At discharge, twenty-five children (38 %) were prescribed stimulant medication and 40 children were not medicated (61 %). One child (2 %) used an antipsychotic drug during the whole study period.

### Inpatient treatment

All participating children were treated at the parent–child ward of the Department of Child and Adolescent Psychiatry and Psychotherapy at the University Hospital of Cologne. The parent–child ward provides intensive treatment that aims at improving parent–child interaction patterns, parenting practices, child problem behaviour and parental strains. Index children are admitted together with their parents and (optionally) siblings. All admitted family members are actively involved in the treatment process. Up to four families are admitted at a time for a four week period. Both parents can be admitted, but children are usually accompanied by their mother only. There are no children on the ward without a parent also being admitted. Most children are referred because of externalizing problem behaviour, but children referred with other diagnoses (e.g., ASD) can also be admitted. All children undergo a structured diagnostic evaluation consisting of psychological and psychiatric assessment, psychological testing (e.g. intelligence), somatic assessment, analysis of the family structure and/or analysis of videotaped parent–child interactions.

The clinical team consists of one senior clinical psychologist (about 30 h/week), three educational staff (full positions), one child and adolescent psychiatrist (about 10 h/week) and two child and adolescent psychotherapists in training (about 20 h/week). They develop an individualized age-appropriate treatment plan for each family that includes child-focused interventions, parent-focused interventions and parent- and child-focused interventions. The psychosocial interventions are based on evidence-based treatment manuals. Since most children are diagnosed with ADHD and/or ODD/CD, the German Treatment Program for Hyperkinetic and Oppositional Problem Behaviour (THOP [20]) is used most frequently. This manual integrates family-based and school-based interventions with cognitive behaviour therapy of the child and has been evaluated in outpatient settings [39]. The family interventions are based on the parent training manuals of Forehand and McMahon [40] and Barkley [41]; the school interventions are based on manuals of DuPaul and Stoner [42] and Swanson [43]. Basic aims and principles for interventions in the family were (1) reduction of specific problem behaviour in specific family or school situations as defined by the Individual Problem Checklist; (2) enhancement of parental attending skills (e.g., during supervised playtime sessions); (3) development of effective methods of communicating commands and setting rules in specific problem situations; (4) implementation of token economies and response cost systems; (5) development of appropriate negative consequences for problem behaviour and use of time out in order to reduce problem behaviour in specific situations (parents and teachers). The child is integrated as an active member in the therapeutic process. The interventions were implemented on the ward and families were taught to use them at home.

Children participate in a daily group training (1.5-2 h/day) that teaches them social and attentional skills (e.g., keeping attention to a task or play activity, social competence training). Individual psychotherapy for the child (e.g., play training, self-instructional training according to THOP [20]) and pharmacological treatment is offered, as indicated by the child’s age and condition. School-aged children attend the school on the hospital grounds. The school provides education for all children receiving inpatient care at the university hospital. In order to meet each child’s requirements, class sizes are small (two to five children) and teachers collaborate closely with the attending physician or psychologist in the inpatient unit (e.g., participate in weekly case conferences, develop an individual treatment plan consistent with the treatment provided at the inpatient unit). Teachers also prepare a detailed report including a recommendation for the most appropriate educational setting after inpatient treatment.

Parents receive group-based behavioural parent training sessions (2 times/week) and individual parent training sessions (2–3 times/week) that target specific behaviour problems of their child. In addition, they are provided with individual psychotherapy as needed (up to 2 or 3 times/week). Parent- and child-focused interventions include dyadic parent–child interaction training consisting of daily positive play time and two weekly coaching sessions. In addition, parents and children participate in weekly parent–child activity groups (e.g., creative activities, excursions) and a weekly therapy session with the whole family (family members that are not admitted to the parent–child ward and/or other professionals involved in the family’s life, such as teachers, youth welfare workers, or psychotherapists may also be invited).

Families spend the weekends at home to generalize new knowledge, skills and behaviour change to the home environment. Special efforts are made to ensure that the families will receive appropriate support after discharge from the parent–child ward. This includes counselling by the clinic’s social service, initiation of youth welfare interventions, initiation of day-treatment programmes, initiation of outpatient psychotherapy for the child and/or the parent, as well as support in selection an appropriate school that meets the individual needs of the child. Table 1 provides an overview of the interventions provided at the parent–child ward.

**Table 1:** Overview of interventions provided at the parent–child ward

	Child-focused interventions	Parent-focused interventions	Parent- and child-focused interventions
Daily	• Group training (social and attentional skills) • School on the hospital grounds (only school-aged children)		• Dyadic parent-child interaction training: positive play time or task focused interaction
Weekly		• Group-based behavioural parent training (2 times/week) • Individual parent training sessions (2-3 times/week)	• Dyadic parent-child interaction training: coaching (2 times/week) • Parent-child activity groups
As indicated	• Individual cognitive-behavioural treatment • Pharmacological treatment • Initiation of youth welfare interventions • Initiation of day- care as follow-up • Initiation of outpatient cognitive-behavioural treatment as follow-up	• Individual psychotherapy • Counselling by the clinic’s social service • Initiation of outpatient psychotherapy as follow-up	• Therapy session with the whole family • Initiation of outpatient psychotherapy as follow-up

### Outcome measures

#### Child Behaviour Checklist (CBCL)

The CBCL 6–18 [38, 44] consists of 113 items that describe typical behavioural and emotional problems. Parents complete each item by answering 0 (*Not true*), 1 (*Somewhat or sometimes true*), or 2 (*Very true or often true*). There are two broad-band syndrome scales (Externalizing and Internalizing Problems) and eight syndrome scales (Withdrawn, Somatic Complaints, Anxious/Depressed, Social Problems, Thought Problems, Attention Problems, Rule-breaking Behaviour and Aggressive Behaviour). Higher scores refer to greater problems. The German version of the CBCL 6–18 has good reliability and validity [44]. Raw scale scores were used in the analysis.

#### Teacher Report Form (TRF)

The TRF 6–18 [38, 44] measures teacher-reported behavioural and emotional problems. The 113 items are rated 0 (*Not true*), 1 (*Somewhat or sometimes true*), or 2 (*Very true or often true*). Analogous to the CBCL, the TRF 6–18 yields two broad-band syndromes and eight syndrome scales. Higher scores refer to greater problems. The German version of the TRF 6–18 has good reliability and validity [44]. Raw scale scores were used in the analysis.

#### ADHD rating scale

The rating scale FBB-ADHS is part of the German ICD- and DSM-based Diagnostic System for the Assessment of Mental Disorders in Children and Adolescents [45]. The FBB-ADHS contains 20 items that assess the occurrence of ADHD symptoms (e.g., “often blurts out answers to questions”) and are rated on four-point Likert scales ranging from 0 (*Not at all*) to 3 (*Very much*). Item scores are averaged to yield a scale score that varies from 0 to 3. Research demonstrated good reliability and validity of FBB-ADHS parent and teacher ratings [46, 47].

#### ODD rating scale

The rating scale FBB-SSV is part of the same diagnostic system as the FBB-ADHS [45]. The scale contains 25 items that are rated from 0 (*Not at all*) to 3 (*Very much*). Nine items corresponded to the symptom criteria for oppositional defiant disorder (ODD) (e.g., “often argues with adults”) and 16 items assessed the symptom criteria for conduct disorder (CD) (e.g., “has broken into someone else's house, building, or car”). Item scores are averaged to yield scale scores that vary from 0 to 3. Only the ODD subscale was used due to the participant’s young age. The FBB-SSV has been found to be a reliable and valid instrument when completed by parents or teachers [48, 49].

#### Individual Problem List (IPL)

The IPL is an individualized outcome measure. With the help of a clinician, parents define four behaviour problems that will be targeted during treatment. Nominated behaviour problems should be either impairing for the child, or stressful for the parent. Parents rate the intensity of each the target behaviour on a four-point Likert scale anchored by 0 (*Not problematic*) and 3 (*Severely disturbing*). The total problem score is derived by averaging the intensity ratings for the target behaviours. Higher scores refer to greater problems.

#### Parent Practices Scale (PPS)

The original version of the PPS [50] contains 34 items and provides scores for two scales, positive and negative parenting practices. In the present study, only the 13-item positive parenting scale was used. Items measure parents' patterns of interaction with their children (e.g., “I praise my child”) on a 4-point scale anchored by 0 (“*Never*”) and 3 (“*Almost always*”). Item scores are averaged to yield a scale score that varies from 0 to 3. A high score refers to positive, reinforcing and supportive parenting behaviour. The German adaptation of the positive parenting practices subscale of the PPS has high internal consistency [26].

#### Self-Efficacy Scale (SEFS)

The SEFS is a German adaptation of the Parenting Sense of Competence Scale [51] and the Self Efficacy for Parenting Task Index [52]. The SEFS comprises 15 items that measure parents’ perception of self-efficacy (e.g., “I meet my own personal expectations for expertise in caring for my child”) on a 4-point scale anchored by 0 (*Does not apply to me at all*) and 3 (*Applies to me much, or most of the time*). Item scores are averaged to yield a scale score that varies from 0 to 3. Higher scores reflect higher self-efficacy. Internal consistency of the German adaptation has been shown to be high [26].

#### Problem Setting and Behaviour Checklist (PSBC)

The PSBC [53] measures parent's belief in their self-efficacy in solving difficult parenting situations, such as shopping with the child or having visitors arrive. The German adaptation of the PSBC contains 27 items that are rated on 4-point scales anchored by 0 (C*ertain I can’t do it*) and 3 (*Certain I can do it*). Item scores are averaged to yield a scale score that varies from 0 to 3. High scores reflect a high ability to deal with difficult parenting situations. The internal consistency of the German adaptation is high [28].

#### Questionnaire on Judging Parental Strains (QJPS)

The QJPS is a 52-item German-language questionnaire that measures the subjective strains of parents of children with ADHD [54]. There are five subscales: Competence and Satisfaction, Solution Orienting, Social Interaction, Partnership and Siblings. Each item (e.g., “My child’s behaviour causes conflict among family members”) is rated on a 4-point scale ranging from 0 (*Does not apply at all/Not distressing*) to 3 (*Very distressing*). Item scores are averaged to yield a scale score that varies from 0 to 3. High scores reflect high strains. Previous studies reported high internal consistency [26].

#### Depression Anxiety Stress Scale (DASS)

The DASS [55] comprises 42 items that assess symptoms of depression, anxiety and stress in adults (e.g., ‘I could see nothing in the future to be hopeful about’). Parents rate the extent to which they have experienced each symptom over the past week on a 4-point severity/frequency scale anchored by 0 (*Did not apply to me at all*) and 3 (*Applied to me much, or most of the time*). Each of the three subscales (Depression, Anxiety, Stress) comprises 14 items. High scores correspond to higher levels of stress, anxiety and/or depression. The internal consistency was shown to be high for all subscales [28].

### Data analysis

Changes in outcomes over time were investigated using multilevel modelling for longitudinal data. Using the 7^th^ version of the HLM software [56], a piecewise growth curve model was specified for each outcome variable. Piecewise growth curve modelling allows for estimation of different growth curves (slopes) for different time periods. Another advantage of growth curve analysis is that it allows for missing data. In the current dataset, no more than two assessment points were missing for a given child on a given rating scale.

The data were analyzed in several steps. First, an intercept-only model (also called null model) was constructed for each outcome variable. Based on the intercept-only model, the intraclass correlation coefficient (ICC) was calculated using the formula: ρ = σ_0_^2^/(σ_0_^2^ + σ_ε_^2^), where σ_0_^2^ represents the level-2 variance component for the intercept and σ_ε_^2^ represents the level-1 residual variance component [57]. The ICC describes the proportion of the total outcome variation that can be explained by differences between individuals. Next, a piecewise growth model was computed for each of the parent-rated outcome variables. Growth rates were calculated for each time period. The growth rate *β*_10_ describes the growth during the waiting period (phase 1). The growth rate *β*_20_ describes the growth during the treatment period (phase 2). The growth rate *β*_30_ describes the growth during the follow-up period (phase 3). A random-intercept model was specified that allows the intercept to vary while growth rates are fixed. We were interested to find out whether the growth rates *β*_20_ (growth during treatment period) and *β*_30_ (growth during follow-up period) are significant, thereby indicating a significant change in the outcome measure after admission to inpatient treatment. In addition, a contrast was defined to determine whether the magnitude of growth rate *β*_20_ (growth during treatment period) is significantly higher than the magnitude of growth rate *β*_10_ (growth during waiting period).

A similar procedure was conducted with the teacher-rated outcome measures. Growth rates were calculated for only two time periods, because teachers did not participate in the post-treatment (T3) assessment. The coefficient *β*_10_ describes the growth between T1 and T2 (phase 1: waiting period). The growth rate *β*_20+30_ describes the growth between T2 and T4 (phase 2 + 3: treatment + follow-up period). Again, random-intercept models are specified that allow the intercept to vary while growth rates are fixed. It was investigated whether the growth rate *β*_20+30_ (growth during treatment + follow-up period) indicates a significant improvement in the outcome measure. In addition, a contrast was defined to determine whether the growth rate *β*_20+30_ has a significantly higher magnitude than the growth rate *β*_10_ (growth during waiting period).

Effect sizes (ES) were calculated for each time period. This was done using the formula: *d* = (*M*_*1*_*–M*_*2*_)*/SD*_*pooled*._. The effect sizes for the PPS, the SEFS and the PSBC were recoded so that a positive value of *d* always indicates an improvement in the outcome measure.

Finally, two sets of additional analyses were conducted. In the first set of analyses, parent ratings (CBCL and ADHD/ODD symptom ratings) were re-analyzed using the same procedure as with teacher ratings. In the second set of additional analyses, we restricted our sample to those children that did not start stimulant medication during the treatment period (*N* = 54). Each piecewise growth model analysis was rerun in this subsample.

## Results

### Parent-rated outcome measures

Table 2 shows means and standard deviations for parent-rated outcome measures at the four measurement times.

**Table 2:** Means, standard deviations and effect sizes for parent-rated outcome measures

		Baseline (T1)		Pre-treatment (T2)		Post-treatment (T3)		Follow-up (T4)		Effect size Cohen’s *d* ^a^		
		*M (SD)*	*n*	*M (SD)*	*n*	*M (SD)*	*n*	*M (SD)*	*n*	Waiting period	Treatment period	Follow-up period
Child behaviour												
	CBCL Total scale	57.32 (25.09)	66	53.98 (24.89)	64	40.06 (24.34)	65	41.01 (25.18)	63	0.13	0.57	−0.04
	CBCL Externalizing	24.05 (10.57)	66	22.69 (10.84)	64	19.53 (10.08)	65	16.88 (9.75)	63	0.13	0.59	−0.04
	CBCL Internalizing	12.74 (7.37)	66	12.06 (6.82)	64	8.68 (6.85)	65	8.96 (7.10)	63	0.10	0.49	−0.04
	ADHD rating scale	1.52 (0.76)	65	1.48 (0.78)	63	1.08 (0.64)	65	1.06 (0.65)	63	0.04	0.57	0.02
	ODD rating scale	1.39 (0.78)	66	1.38 (0.74)	65	0.99 (0.62)	65	0.96 (0.55)	63	0.01	0.57	0.05
	IPL	2.59 (0.41)	63	2.11 (0.73)	66	1.27 (0.58)	66	1.22 (0.60)	60	0.81	1.27	0.09
Parenting, parental strains, and parental mental health												
	PPS	1.93 (0.45)	66	1.85 (0.48)	65	2.08 (0.40)	66	2.00 (0.45)	63	−0.17^b^	0.50^b^	−0.17^b^
	SEFS	1.78 (0.56)	66	1.78 (0.49)	64	2.01 (0.44)	66	2.07 (0.42)	63	0.00^b^	0.49^b^	0.13^b^
	PSBC	2.90 (0.53)	66	2.90 (0.49)	64	3.11 (0.47)	66	3.27 (0.41)	63	0.01^b^	0.44^b^	0.35^b^
	QJPS	1.30 (0.79)	66	1.28 (0.73)	64	0.82 (0.71)	65	0.77 (0.60)	63	0.03	0.65	0.07
	DASS	1.90 (0.65)	65	2.02 (0.64)	65	1.54 (0.49)	66	1.59 (0.54)	60	−0.18	0.84	−0.08

*CBCL* Child Behaviour Checklist, *IPL* Individual Problem Checklist, *PPS* Parent Practices Scale, *SEFS* Self-Efficacy Scale, *PSBC* Problem Setting and Behaviour Checklist, *QJPS* Questionnaire on Judging Parental Strains, *DASS* Depression Anxiety Stress Scale

^a^Effect sizes were calculated using the formula: *d* = (*M*_*1*_ *– M*_*2*_)*/SD*_*pooled*_

^b^Effect sizes were recoded so that positive values indicate improvement

#### Intraclass correlations (ICC)

The ICC for the parent-rated outcome measures are ρ = 0.72 for CBCL Total scale, *ρ* = 0.66 for CBCL Externalizing scale, ρ = 0.72 for CBCL Internalizing scale, ρ = 0.62 for ADHD symptom ratings, ρ = 0.56 for ODD symptom ratings, ρ = 0.66 for PPS, ρ = 0.64 for SEFS, ρ = 0.43 for PSBC, ρ = 0.61 for QJPS and ρ = 0.50 for the DASS. This indicates that, for most variables, more than half of the total variance can be attributed to differences between individuals and less than half of the variance is explained by differences within individuals across time. The ICC for the IPL is ρ = 0.0006, indicating that 99 % of the variance in IPL scores can be explained by differences within individuals across time.

#### Piecewise linear growth models

The upper part of Table 3 presents the results of the growth model analyses conducted on parent-rated child behaviour measures. The growth rate *β*_10_ was significant for the IPL (*t*_(186)_ = −5.19, *p* < .001), indicating a significant decrease in scores (improvement) during the waiting period. There was no significant change in scores during the waiting period on the CBCL Total scale (*t*_(189)_ = −1.75, *p* = .082), the CBCL Externalizing scale (*t*_(189)_ = −1.43, *p* = .154), the CBCL Internalizing scale (*t*_(189)_ = −1.29, *p* = .199), the ADHD symptom rating scale (*t*_(187)_ = −0.24, *p* = .812) and the ODD symptom rating scale (*t*_(190)_ = −0.21, *p* = .833).

**Table 3:** Results of piecewise linear growth models for parent-rated outcome measures

		Intercept	*β*_10_	*β*_20_	*β*_30_	*β*_10_ vs. *β*_20_
			(waiting period)	(treatment period)	(follow-up period)	(contrast)
Child behaviour						
	CBCL Total scale	57.32***	−3.18	−13.90***	−0.04	*β*_10_ < *β*_20_**
	CBCL Externalizing	24.05***	−1.25	−6.08***	−0.14	*β*_10_ < *β*_20_**
	CBCL Internalizing	12.74***	−0.74	−3.31***	0.10	*β*_10_ < *β*_20_**
	ADHD rating scale	1.50***	−0.02	−0.40***	−0.04	*β*_10_ < *β*_20_**
	ODD rating scale	1.39***	−0.02	−0.37***	−0.06	*β*_10_ < *β*_20_**
	IPL	2.58***	−0.48***	−0.84***	−0.06	*β*_10_ < *β*_20_*
Parenting, parental strains, and parental mental health						
	PPS	1.93***	−0.08	0.23***	−0.07	*β*_10_ < *β*_20_***
	SEFS	1.78***	−0.01	0.24***	0.07	*β*_10_ < *β*_20_**
	PSBC	2.90***	0.002	0.21***	0.17**	*β*_10_ < *β*_20_*
	QJPS	1.30***	−0.05	−0.43***	−0.09	*β*_10_ < *β*_20_**
	DASS	1.90***	0.12*	−0.47***	0.02	*β*_10_ < *β*_20_***

*CBCL* Child Behaviour Checklist, *IPL* Individual Problem Checklist, *PPS* Parent Practices Scale, *SEFS* Self-Efficacy Scale, *PSBC* Problem Setting and Behaviour Checklist, *QJPS* Questionnaire on Judging Parental Strains, *DASS* Depression Anxiety Stress Scale

*p < .05, **p < .01, ***p > .001

The growth rate *β*_20_ was significant for all parent-rated child behaviour measures, indicating a significant decrease in scores (improvement) on each of these measures during the treatment period (CBCL Total scale: *t*_(189)_ = −7.62, *p* < .001; CBCL Externalizing scale: *t*_(189)_ = −6.92, *p* < .001; CBCL Internalizing scale: *t*_(189)_ = −5.77, *p* < .001; ADHD rating scale: *t*_(187)_ = −5.86, *p* < .001; ODD rating scale: *t*_(190)_ = −5.08, *p* < .001; IPL: *t*_(186)_ = −9.25, *p* < .001). None of these variables showed a significant change in scores during the follow-up period (*β*_30_), indicating that improvements in child behaviour were maintained at follow-up (CBCL Total scale: *t*_(189)_ = −0.02, *p* = .983; CBCL Externalizing scale: *t*_(189)_ = −0.16, *p* = .874; CBCL Internalizing scale: *t*_(189)_ = 0.18, *p* = .861; ADHD rating scale: *t*_(187)_ = −0.56, *p* = .576; ODD rating scale: *t*_(190)_ = −0.80, *p* = .425; IPL: *t*_(186)_ = −0.70, *p* = .485).

**Fig. 2:**
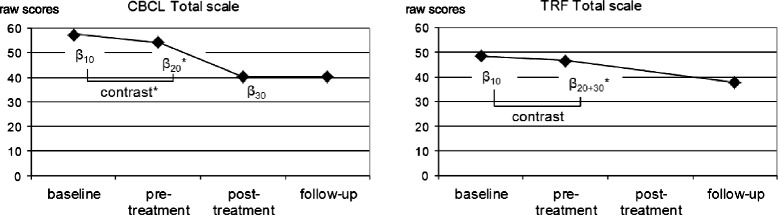
Trajectory of raw scores on the Child Behaviour Checklist (CBCL) Total scale and the Teacher Report Form (TRF) Total scale for a prototypical individual based on parameters estimated by piecewise linear growth modeling. *p < .05

**Fig. 3:**
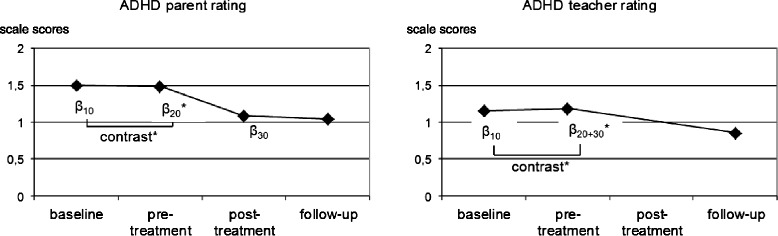
Trajectory of raw scores on the ADHD symptom rating scale for a prototypical individual based on parameters estimated by piecewise linear growth modeling. *p < .05

**Fig. 4:**
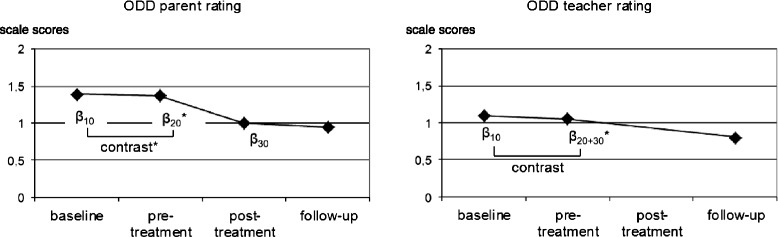
Trajectory of raw scores on the ODD symptom rating scale for a prototypical individual based on parameters estimated by piecewise linear growth modeling. *p < .05

Contrasts indicated that the magnitude of the growth rate *β*_20_ was significantly higher than the magnitude of the growth rate *β*_10_ for all parent-rated child behaviour measures (CBCL Total scale: *χ*^2^_(1)_ = 11.52, *p* = .001; CBCL Externalizing scale: *χ*^2^_(1)_ = 10.07, *p* = .002; CBCL Internalizing scale: *χ*^2^_(1)_ = 6.72, *p* = .009; ADHD rating scale: *χ*^2^_(1)_ = 10.47, *p* = .002; ODD rating scale: *χ*^2^_(1)_ = 7.94, *p* = .005; IPL: *χ*^2^_(1)_ = 5.27, *p* = .020). Figures 2, 3 and 4 (left part) display the results for the CBCL Total scale, the ADHD rating scale and the ODD/CD rating scale.

The lower part of Table 3 presents the results of the growth model analyses conducted on the outcome measures assessing parenting, parental strains and parental mental health. The growth rate *β*_10_ was significant for the DASS scale (*t*_(187)_ = 2.09, *p* = .038), indicating a significant worsening in parental mental health during the waiting period. There was no significant change in scores on the PPS (*t*_(191)_ = −1.94, *p* = .054), the SEFS (*t*_(190)_ = −0.13, *p* = .894), the PSBC (*t*_(190)_ = 0.04, *p* = .968), or the QJPS (*t*_(189)_ = −0.74, *p* = .459), indicating that parenting skills and parental strains were stable during the waiting period.

The growth rate *β*_20_ was significant for all outcome measures assessing parenting, parental strains and parental mental health, indicating a significant improvement on each of these measures during the treatment period (PPS: *t*_(191)_ = 5.22, *p* < .001; SEFS: *t*_(190)_ = 5.27, *p* < .001; PSBC: *t*_(190)_ = 3.65, *p* < .001; QJPS: *t*_(189)_ = −6.63, *p* < .001; DASS: *t*_(187)_ = −8.462, *p* < .001). Scores on the PSBC improved significantly from post-treatment to follow-up (*t*_(190)_ = 2.91, *p* = .004). None of the remaining measures showed a significant change in scores during the follow-up period, indicating that improvements were maintained at follow-up (PPS: *t*_(191)_ = −1.52, *p* = .131; SEFS: *t*_(190)_ = 1.64, *p* = .103; QJPS: *t*_(189)_ = −1.31, *p* = .191; DASS: *t*_(187)_ = 0.36, *p* = .723). Significant contrasts indicated that the growth rate *β*_20_ was significantly higher than the growth rate *β*_10_ for all outcome measures assessing parenting, parental strains and parental mental health (PPS: *χ*^2^_(1)_ = 17.00, *p* < .001; SEFS: *χ*^2^_(1)_ = 9.66, *p* = .002; PSBC: *χ*^2^_(1)_ = 4.31, *p* = .036; QJPS: *χ*^2^_(1)_ = 11.56, *p* = .001; DASS: *χ*^2^_(1)_ = 36.27, *p* < .001).

#### Effect sizes

The effect sizes for change during the waiting period (T1–T1) are small (*d* = −0.18 – 0.13), except for the IPL (*d* = 0.81), indicating that most variables showed little change during the waiting period (see Table 2). Medium to large effect sizes (*d* = 0.44 – 1.27) were obtained for change during inpatient treatment (T2 – T3), while small effect sizes (*d* = −0.17 – 0.35) were found for change during follow-up (T3–T4).

### Teacher-rated outcome measures

Table 4 shows means and standard deviations and effect sizes for teacher-rated outcome measures at the three measurement times.

**Table 4:** Means, standard deviations and effect sizes for teacher-rated outcome measures

		Baseline (T1)		Pre-treatment (T2)		Follow-up (T4)		Effect size Cohen’s *d* ^a^	
		*M (SD)*	*n*	*M (SD)*	*n*	*M (SD)*	*n*	Waiting period	Treatment + follow-up period
Child behavior									
	TRF Total scale	48.40 (27.48)	56	46.71 (26.95)	55	37.75 (27.13)	55	0.06	0.33
	TRF Externalizing	19.99 (12.63)	56	19.12 (12.44)	55	14.67 (12.56)	55	0.07	0.36
	TRF Internalizing	7.43 (5.95)	56	6.79 (5.32)	55	6.82 (6.05)	55	0.11	−0.005
	ADHD rating scale	1.13 (0.79)	54	1.19 (0.81)	54	0.85 (0.76)	55	−0.07	0.43
	ODD rating scale	1.09 (0.77)	53	1.05 (0.95)	53	0.80 (0.72)	53	0.04	0.30

*TRF* Teacher Report Form

^a^Effect sizes were calculated using the formula: *d* = (*M*_*1*_ *– M*_*2*_)*/SD*_*pooled*_

#### Intraclass correlations (ICC)

The intraclass correlations (ICC) for the teacher-rated outcome measures are ρ = 0.69 for TRF Total scale, ρ = 0.69 for TRF Externalizing scale, ρ = 0.64 for TRF Internalizing scale, ρ = 0.74 for ADHD symptom ratings and ρ = 0.60 for ODD symptom ratings. These values indicate that more than half of the total variance can be attributed to differences between individuals.

#### Piecewise linear growth models

Table 5 presents the results of the growth model analyses conducted on teacher-rated child behaviour measures. The growth rate *β*_10_ was not significant for any for the teacher-completed rating scales, indicating that scores did not change significantly during the waiting period (TRF Total scale: *t*_(107)_ = −0.72, *p* = .474; TRF Externalizing scale: *t*_(107)_ = −0.87, *p* = .389; TRF Internalizing scale: *t*_(107)_ = −0.79, *p* = .430; ADHD rating scale: *t*_(104)_ = 0.45, *p* = .651; ODD rating scale: *t*_(100)_ = −0.43, *p* = .665),

**Table 5:** Results of piecewise linear growth models for teacher-rated outcome measures

		Intercept	*β*_10_	*β*_20+30_	*β*_10_ vs. *β*_20+30_
			(waiting period)	(treatment + follow-up period)	(contrast)
Child behaviour					
	TRF Total scale	48.49***	−1.94	−8.89**	*β*_10_ = *β*_20+30_
	TRF Externalizing	20.07***	−1.07	−4.52***	*β*_10_ = *β*_20+30_
	TRF Internalizing	7.39***	−0.53	0.08	*β*_10_ = *β*_20+30_
	ADHD rating scale	1.15***	0.03	−0.33***	*β*_10_ < *β*_20+30_**
	ODD rating scale	1.09***	−0.04	−0.25*	*β*_10_ = *β*_20+30_

*TRF* Teacher Report Form

*p < .05, **p < .01, ***p > .001

The growth rate *β*_20+30_ was significant for all teacher-rated child behaviour measures, except for the TRF Internalizing scale, indicating a significant decrease in scores (improvement) on most measures during the treatment + follow-up period (TRF Total scale: *t*_(107)_ = −3.27, *p* = .001; TRF Externalizing scale: *t*_(107)_ = −3.63, *p* < .001; TRF Internalizing scale: *t*_(107)_ = 0.12, *p* = .905; ADHD rating scale: *t*_(104)_ = −4.59, *p* < .001; ODD rating scale: *t*_(100)_ = −2.48, *p* = .015). A pre-defined contrast revealed that the magnitude of the growth rate *β*_20+30_ was significantly higher than the magnitude of the growth rate *β*_10_ for the teacher-completed ADHD symptom rating scale (*χ*^2^_(1)_ = 8.49, *p* = .004). There was no significant difference between growth rate *β*_20+30_ and growth rate *β*_10_ for any of the other teacher-completed rating scales (TRF Total scale: *χ*^2^_(1)_ = 2.19, *p* = .135; TRF Externalizing Scale: *χ*^2^_(1)_ = 2.57, *p* = .105; TRF Internalizing scale: *χ*^2^_(1)_ = 0.28, *p* > .50; ODD rating scale: *χ*^2^_(1)_ = 1.42, *p* = .231). Figures 2, 3 and 4 (right part) display the results for the TRF Total scale, the ADHD rating scale and the ODD rating scale.

#### Effect sizes

All effect sizes for change during the waiting period (T1–T2) are small (*d* = −0.07 – 0.11), indicating that teacher-rated child behaviour was stable during the waiting period (see Table 3). Effect sizes for change during the treatment + follow-up period (T2–T4) were small to medium (*d* = 0.30 – 0.43), except for the TRF Internalizing scale (*d* = −0.005).

### Additional analyses

In the first set of additional analyses, parent ratings were re-analyzed using the same procedure as with teacher ratings. This was done to be better able to compare results obtained from parent and teacher ratings. Two growth rates were calculated for each measure. The coefficient *β*_10_ describes the growth between T1 and T2 (phase 1: waiting period) and *β*_20+30_ describes the growth between T2 and T4 (phase 2 + 3: treatment + follow-up period).

The growth rate *β*_10_ was significant for the CBCL Total scale (*β*_10_ = −3.11, *t*_(125)_ = −1.73, *p* = .086), but not for the CBCL Externalizing scale (*β*_10_ = −1.20, *t*_(125)_ = −1.36, *p* = .177), the CBCL Internalizing scale (*β*_10_ = −0.73, *t*_(125)_ = −1.29, *p* = .198), the ADHD symptom rating scale (*β*_10_ = −0.01, *t*_(123)_ = −0.18, *p* = .859) and the ODD symptom rating scale (*β*_10_ = −0.01, *t*_(126)_ = −0.195, *p* = .846). The growth rate *β*_20+30_ was significant for all measures, indicating a significant decrease in scores (improvement) during the treatment + follow-up period (CBCL Total scale: *β*_20+30_ = −13.74, *t*_(125)_ = −7.52, *p* < .001; CBCL Externalizing scale: *β*_20+30_ = −6.16, *t*_(125)_ = −6.84, *p* < .001; CBCL Internalizing scale: *β*_20+30_ = −3.15, *t*_(125)_ = −5.52, *p* < .001; ADHD symptom rating: *β*_20+30_ = −0.44, *t*_(123)_ = −6.49, *p* < .001; ODD symptom rating: *β*_20+30_ = −0.42, *t*_(126)_ = −5.43, *p* < .001). Pre-defined contrasts revealed that the magnitude of the growth rate *β*_20+30_ was significantly higher than the magnitude of the growth rate *β*_10_ for CBCL Total scale (*χ*^2^_(1)_ = 11.44, *p* = .001), the CBCL Externalizing scale (*χ*^2^_(1)_ = 10.25, *p* = .002), the CBCL internalizing scale (*χ*^2^_(1)_ = 6.11, *p* = .013), the ADHD symptom rating scale (*χ*^2^_(1)_ = 13.36, *p* = .001) and the ODD symptom rating scale (*χ*^2^_(1)_ = 9.30, *p* = .003). The results of this set of additional analyses confirm our previous observation that parent ratings of child behaviour improved significantly more during the treatment period than during the pre-admission waiting period.

The second set of additional analyses was conducted on the subsample of children that did not start stimulant medication during the treatment period (*N* = 54). Table 6 displays the results for the parent-rated outcome measures. For most variables, the results are consistent with those obtained in the whole sample. For the CBCL Internalizing scale and the IPL, the pre-defined contrast (*β*_10_ vs. *β*_20_) was no longer statistically significant. Since the growth rates *β*_10_ and *β*_20_ differ only slightly in the whole sample and the subsample (by 0.05 – 0.4), the failure to find significant contrasts in the subsample is probably due to a reduction of statistical power. Table 7 displays the results for the teacher-rated outcome measures. Again, the results are largely consistent with those obtained in the whole sample, with only few exceptions. In the reduced sample, the growth rate *β*_20+30_ (growth during treatment + follow-up period) was no longer significant for the ODD rating scale, but the pre-defined contrast (*β*_10_ vs. *β*_20+30_) was still significant. Again, the growth rate differs only slightly between the whole sample (*β*_20+30_ = −0.25) and the subsample (*β*_20+30_ = −0.17), suggesting that the failure to reach statistical significance resulted from a reduction of statistical power.

**Table 6:** Results of piecewise linear growth models for parent-rated outcome measures (*N* = 54, after excluding children who started stimulant medication during the treatment period)

		Intercept	*β*_10_	*β*_20_	*β*_30_	*β*_10_ vs. *β*_20_
			(waiting period)	(treatment period)	(follow-up period)	(contrast)
Child behaviour						
	CBCL Total scale	57.45***	−2.86	−12.97***	0.02	*β*_10_ < *β*_20_**
	CBCL Externalizing	23.76***	−0.92	−5.62***	0.07	*β*_10_ < *β*_20_**
	CBCL Internalizing	12.84***	−0.79	−2.92***	0.07	*β*_10_ = *β*_20_
	ADHD rating scale	1.41***	−0.003	−0.31***	−0.03	*β*_10_ < *β*_20_*
	ODD rating scale	1.31***	0.02	−0.32***	−0.06	*β*_10_ < *β*_20_**
	IPL	2.57***	−0.52***	−0.74***	−0.07	*β*_10_ = *β*_20_
Parenting, parental strains and parental mental health						
	PPS	1.93***	−0.08	0.23***	−0.05	*β*_10_ < *β*_20_***
	SEFS	1.78***	0.02	0.21***	0.08	*β*_10_ < *β*_20_*
	PSBC	2.92***	−0.01	0.25***	0.13*	*β*_10_ < *β*_20_**
	QJPS	1.25***	−0.09	−0.40***	−0.03	*β*_10_ < *β*_20_**
	DASS	1.89***	0.05	−0.44***	0.04	*β*_10_ < *β*_20_***

*CBCL* Child Behaviour Checklist, *IPL* Individual Problem Checklist, *PPS* Parent Practices Scale, *SEFS* Self-Efficacy Scale, *PSBC* Problem Setting and Behaviour Checklist, *QJPS* Questionnaire on Judging Parental Strains, *DASS* Depression Anxiety Stress Scale

*p < .05, **p < .01, ***p > .001

**Table 7:** Results of piecewise linear growth models for teacher-rated outcome measures (*N* = 47, after excluding children who started stimulant medication during the treatment period)

		Intercept	*β*_10_	*β*_20+20_	*β*_10_ vs. *β*_20+30_
			(waiting period)	(treatment + follow-up period)	(contrast)
Child behaviour					
	TRF Total scale	46.30***	−0.86	−6.40*	*β*_10_ = *β*_20+30_
	TRF Externalizing	18.81***	−0.74	−3.12*	*β*_10_ = *β*_20+30_
	TRF Internalizing	7.64***	−0.42	−0.08	*β*_10_ = *β*_20+30_
	ADHD rating scale	1.07***	0.05	−0.23**	*β*_10_ < *β*_20+30_*
	ODD rating scale	1.01***	−0.02	−0.17	*β*_10_ = *β*_20+30_

*TRF* Teacher Report Form

*p < .05, **p < .01, ***p > .001

## Discussion

This study evaluated a parent–child inpatient setting that provides intensive treatment to children and their parents (primarily mothers) based on evidence-based treatment manuals. The use of a within-subjects design with four assessment points enabled us to compare changes in outcomes during a four-week pre-admission waiting period, a four-week treatment period and a four-week follow-up interval. Child behaviour was assessed by parent and teacher ratings on the Achenbach scales (CBCL/TRF) and ADHD/ODD symptom rating scales according to ICD-10 and DSM-IV. Parents also rated the intensity of four individually defined child behaviour problems that they wished to alter during treatment (Individual Problem List, IPL). Further outcome variables were self-rating scales for parenting, parental strains and parental mental health.

Our results show that child behaviour problems, parenting practices, parental strains and parental mental health were stable during the waiting period. Only scores on the Individual Problem List (IPL) decreased significantly between baseline and admission (*d* = 0.81), indicating that the individual target behaviour improved during the pre-admission waiting period. This finding suggests that establishing explicit treatment goals with the parent is an essential component of parent training and may, by itself, lead to improvement. None of the other outcome measures changed significantly during the pre-admission period (*d* < 0.2).

Consistent with our expectations, all parent-rated measures improved significantly during the treatment period. The effect sizes were medium to large, with the largest effect size for the individually defined target behaviour (IPL: *d* = 1.27; other child behaviour measures*: d* = 0.49 – 0.59; parenting: *d* = 0.44 – 0.50; parental strains: *d* = 0.65; parental mental health: *d* = 0.84). Significant contrasts indicated that all parent-rated measures improved more during treatment than during the waiting period. This finding supports the hypothesis that changes observed during the treatment period result from parent–child inpatient treatment and are unlikely to be caused by the mere passage of time or repeated assessment. In addition, we found that treatment gains were maintained at the four-week follow-up. None of the parent-rated measures changed significantly during the follow-up period (*d* < 0.2), except for parents’ belief in their self-efficacy in solving difficult parenting situations (PSBC), which showed continued improvement (*d* = 0.35).

Teachers rated children’s behaviour at baseline, at admission and at the four-week follow-up. They were not asked to provide ratings at post-intervention because the children did not attend their regular school during their stay at the parent–child ward. None of the outcome measures rated by teachers changed significantly between baseline and admission (*d* < 0.12), indicating that children’s behaviour at school was stable during the pre-admission waiting period. In line with our expectations, teacher ratings on the TRF Externalizing scale and the ADHD/ODD rating scales improved significantly between admission and follow-up (*d* = 0.30 – 0.43). Scores on the teacher-completed ADHD rating scale improved significantly more during the treatment + follow-up period than during the pre-admission waiting period. Other contrasts were not significant, although improvements in teacher-rated externalizing behaviour and ODD symptoms were substantially larger during the treatment + follow-up period than during the waiting period. Based on these findings, we conclude that the parent–child inpatient treatment had a positive effect on children’s disruptive behaviour at school. There was no treatment-related effect on teacher evaluations of children’s internalizing behaviour.

Only a few studies have previously reported on the effects of inpatient treatment for the whole family in a child psychiatric setting. They provided evidence that family inpatient treatment positively influences parent evaluations of their children’s behaviour, their family climate, their parenting skills and their distress [35–37]. Consistent with these results, we found that parent–child inpatient treatment improves children’s behaviour and leads to improved scores on parents’ self-ratings of their parenting skills, their strains and their mental health. The present study extends the existing knowledge base in two important ways. First, it is the first study to show that parent–child inpatient treatment not only improves parent ratings of child behaviour, but also positively influences children’s behaviour at school. Second, our results, to our knowledge, are the first demonstration that treatment effects are maintained at follow up, after the families had returned to their homes.

Despite the advantages of our design, the study is still subject to some limitations. It should, for example, be mentioned that 38 % of the sample left the hospital with a prescription of stimulant medication. To determine the extent to which the onset of medication during the inpatient treatment influenced our findings, additional analyses were conducted on the subsample of children that did not start medical treatment (*N* = 54) during the treatment period. This is a conservative approach for data analysis because it includes children that stopped taking medication during the trial. The results were largely consistent with those obtained in the whole sample, suggesting that the start of medication is not the main component of the treatment effects observed in this study.

Another limitation that should be noted is the lack of an untreated control group which was considered unethical for these families. We addressed this limitation by using a within-subjects design with four assessment points. This approach enabled us to test for differences between the four-week pre-admission waiting period and the four-week treatment period. All parent-rated measures improved significantly more during the treatment period than during the waiting period. Teacher-rated disruptive child behaviour also improved substantially more during treatment. We therefore conclude that the observed benefits result from parent–child inpatient treatment and cannot be explained by non-specific effects such as repeated testing or spontaneous recovery. Other non-specific effects, such as placebo, cannot be ruled out with the current design.

For practical reasons, it was not possible to include an outpatient control group with comparable treatment intensity. The study, therefore, does not allow firm conclusions regarding the importance of admitting families with severe parent–child interaction problems to an inpatient unit. An outpatient treatment programme with the same amount of parent-focused, child-focused and parent–child-focused interventions might lead to similar treatment effects. However, attendance rates for outpatient parent training programmes are often low [30, 31] and the treatment intensity provided at the parent–child ward might be difficult to realize in an outpatient setting.

It should also be mentioned that a wide array of additional individualised services (e.g., initiation of youth welfare) was part of the inpatient treatment and implemented in a minority of patients. Nevertheless, it is difficult to disentangle the effects of these services from those of the inpatient treatment per se.

A common limitation of intervention studies is the lack of blinded outcome assessment [10] which may cause expectancy effects. Parents could not be blinded because they took an active part in the intervention. This may lead to bias, especially when parents participate in inpatient treatment programmes where they might be more committed to the treatment compared with outpatient services. However, unblinded effects also cover nonspecific effects during treatment (e.g., change in parental expectations) which were also important components of the treatment. In addition, there is evidence from an effectiveness study of a parent management training that treatment effects are not strongly biased due to participation in the training [58]. Schools had to be informed about the treatment because all children in this study were of compulsory school age. Although teachers were not blinded and may have expectations about the change that has been accomplished during the child’s absence, their ratings are a less proximal measurement and are less likely to be influenced by social desirability response bias. The reliance on unblinded parents’ and teachers’ reports as the only source of outcome data is nevertheless a potential limitation and we cannot preclude the possibility that ratings may have been influenced by expectancy effects. The use of more objective observational measures, such as videotaped interactions scored by blinded raters, and the assessment of the long-term stability of treatment effects should help to overcome this limitation in future research.

It should further be mentioned that the lack of formal treatment fidelity checks might affect the internal validity of our findings. However, the treatment team was supervised on a weekly basis including treatment integrity checks. Lastly, we would like to note that we did not explore possible moderators of the treatment effect. It is, therefore, not clear whether the treatment provided at the parent–child ward is equally effective across age groups.

## Conclusions

Our findings show that parent–child inpatient treatment in a child psychiatric setting is an effective treatment for children with behavioural problems and severe parent–child interactions problems. The study adds to and extends the emerging literature on parent–child inpatient treatment [35–37]. The predominant approach in child and adolescent psychiatry is to admit children without their parents. As studies have shown, better outcome of child psychiatric inpatient treatment is predicted by better family functioning at admission and positive parental therapeutic alliance [3]. However, the family’s ability to contain the child is often considered for admission decisions [59] and most parents attend relatively few parent sessions [6, 7]. The present study provides evidence that families with severe parent–child interaction problems benefit from inpatient treatment of parent–child interaction problems that includes parent-focused, child-focused and parent–child-focused interventions.
